# Combined Targeting of PD-1 and TIM-3 in Patients with Locally Advanced or Metastatic Melanoma: AMBER Cohorts 1c, 1e, and 2A

**DOI:** 10.1158/1078-0432.CCR-25-0884

**Published:** 2025-06-24

**Authors:** Diwakar Davar, Zeynep Eroglu, Casilda Llácer Pérez, Brian Di Pace, Tianli Wang, Niranjan Yanamandra, Srimoyee Ghosh, Kathrin Jansen, Arindam Dhar, Theo Borgovan, Angela Waszak, Antoni Ribas

**Affiliations:** 1 Division of Hematology/Oncology, Department of Medicine, Hillman Cancer Center, University of Pittsburgh Medical Center, Pittsburgh, Pennyslvania.; 2 Department of Cutaneous Oncology, Moffitt Cancer Center, Tampa, Florida.; 3 Medical Oncology Intercenter Unit, Regional and Virgen de la Victoria Hospitals, IBIMA Plataforma BIONAND, Málaga, Spain.; 4 GSK, Collegeville, Pennyslvania.; 5 GSK, Waltham, Massachusetts.; 6 GSK, Heidelberg, Germany.; 7 Jonsson Comprehensive Cancer Center, University of California, Los Angeles, Los Angeles, California.

## Abstract

**Purpose:**

The phase I, open-label, multicenter AMBER study (NCT02817633) is evaluating cobolimab, an anti–T-cell immunoglobulin and mucin-domain containing protein-3 humanized mAb, as a monotherapy and combination therapy in patients with solid tumors. In this study, the safety and efficacy of cobolimab plus dostarlimab, a PD-1 inhibitor, in patients with locally advanced/metastatic melanoma who were either immunotherapy-naïve or had progressed on prior anti–PD-(L)1 therapy, are reported.

**Patients and Methods:**

Adults with adequate organ function and either immunotherapy-naïve (parts 1c/1e) or anti–PD-(L)1 relapsed or refractory (part 2A) melanoma were enrolled and received cobolimab 100, 300, or 900 mg and dostarlimab 500 mg every 3 weeks. Treatment continued until disease progression, unacceptable toxicity, withdrawal of consent, or death (whichever occurred sooner). Endpoints included safety, tolerability, overall response rate, and disease control rate.

**Results:**

The current integrated analysis included 28 patients who received treatment in parts 1c/1e and 43 patients who received treatment in part 2A. Treatment-related serious adverse events were observed in 14.3% and 9.3% of patients in parts 1c/1e and 2A, respectively. The overall response rate (95% confidence interval) was 42.9% (24.5–62.8) and 4.7% (0.6–15.8) for patients in parts 1c/1e and 2A, respectively, and the disease control rate (95% confidence interval) was 53.6% (33.9–72.5; 1c/1e) and 20.9% (10.0–36.0; 2A).

**Conclusions:**

In this exploratory setting, cobolimab plus dostarlimab was well tolerated, with reported preliminary efficacy similar to other anti–T-cell immunoglobulin and mucin-domain containing protein-3 treatments in patients with locally advanced/metastatic melanoma.

*See related article by Davar et al., p. 3443*

**Translational Relevance**

Co-blockade of PD-(L)1 and T-cell immunoglobulin and mucin domain–containing protein-3 has been shown to improve antitumor T-cell responses both *in vitro* and in patients with solid tumors. The AMBER study is evaluating the safety and efficacy of the anti–T-cell immunoglobulin and mucin-domain containing protein-3 humanized mAb cobolimab in combination with the PD-1 inhibitor dostarlimab. The current analysis assessed patients with locally advanced/metastatic melanoma who were either immunotherapy-naïve (parts 1c/1e) or anti–PD-(L)1 relapsed or refractory (part 2A). The results demonstrated that the combination was well-tolerated and showed preliminary efficacy in a subset of patients. Based on these findings, further investigations of combined dual checkpoint inhibition with cobolimab plus dostarlimab in solid tumors are warranted.

## Introduction

The use of mAbs to target the immunoregulatory checkpoint proteins programmed cell death protein 1 (PD-1), cytotoxic T-lymphocyte–associated protein-4 (CTLA-4), or lymphocyte-activation gene-3 (LAG-3) have transformed the management of advanced melanoma (1–3). These immune checkpoint inhibitor (ICI) therapies are associated with an overall response rate (ORR) of up to 55% in patients with previously untreated, unresectable stage III or IV melanoma, with a durable response observed for many patients during extended follow-up (3–7). Kinase inhibitors targeting oncogenic v-Raf murine sarcoma viral oncogene homolog B1 (*BRAF*) genes are also associated with a high ORR and improved survival for patients with advanced melanoma when received as a monotherapy or in combination with mitogen-activated protein kinase (MEK) inhibitors (8). Although triplet combinations of BRAF and MEK inhibitors with PD-(L)1 inhibitors are approved for use in this patient population, these combinations have demonstrated high incidences of treatment-related adverse events (TRAE) compared with control patients (9, 10).

Studies assessing comparisons of treatment sequencing approaches have demonstrated that, in patients with *BRAF* mutations, upfront immunotherapy is superior to BRAF/MEK targeted therapy (11–13); however, the optimal therapeutic approach for patients who progress on upfront anti–PD-(L)1 therapy is unclear. Combined PD-1 blockade with either standard or low dose of CTLA-4 inhibitor ipilimumab (3 mg/kg or 1 mg/kg once every 3 weeks for four doses) has been associated with an ORR of 28% to 29% (14, 15), highlighting an unmet treatment need and potential course of action for these patients.

T-cell immunoglobulin and mucin-domain containing protein-3 (TIM-3) is an immunoregulatory checkpoint receptor widely expressed in adaptive and innate immune cell types, including NK cells and monocytes (16–21), with emerging data suggesting that TIM-3 functions as a key inhibitory receptor in myeloid cells, particularly macrophages and dendritic cells (DC; ref. 22). TIM-3 is expressed on CD4^+^ T helper and CD8^+^ cytotoxic T cells, particularly a terminally exhausted or dysfunctional subset of these cells (16), and has been identified as an important negative regulator of antitumor immunity across several solid tumors (17–19, 23).

Co-blockade of TIM-3 and PD-(L)1 has been shown to reduce tumor progression in animal models and improve antitumor T-cell responses in patients with cancers, including those with melanoma (17, 18, 21, 24). Moreover, *in vitro* models have demonstrated that co-blockade of TIM-3 and PD-(L)1 was more effective at reducing tumor growth than blocking either pathway alone (18). Overall, these data suggest that TIM-3 acts on both T cells and myeloid cells within tumors to mediate immune suppression.

Cobolimab (TSR-022/GSK4069889) is an anti–TIM-3 humanized mAb, which may act by inhibiting the interaction between TIM-3 and phosphatidylserine (25) and binding with high affinity to TIM-3, resulting in enhanced T-cell activity in *ex vivo* T-cell stimulation assays (26). Dostarlimab is an anti–PD-1 mAb that has demonstrated activity in multiple solid tumors (27, 28). AMBER (NCT02817633) is a two-part (dose escalation and expansion) phase I study evaluating cobolimab as monotherapy and in combination with other drugs in advanced solid tumors (29). Initial results demonstrated that cobolimab in combination with dostarlimab had an acceptable safety profile and was associated with antitumor activity in patients with solid tumors, including advanced or metastatic non–small cell lung cancer (NSCLC) and melanoma, who had progressed after previous treatment or were intolerant to standard treatment (29, 30). In this study, the combination of cobolimab and dostarlimab in patients with locally advanced or metastatic melanoma is reported, including for patients not previously treated with anti–PD-1 therapy (i.e., PD-1–naïve; parts 1c/1e) and patients whose disease had progressed on prior anti–PD-(L)1 treatment (part 2A).

## Patients and Methods

### Study design and conduct

AMBER is an ongoing study being conducted in accordance with ethical principles founded in the Declaration of Helsinki in accordance with the protocol. All study information that required preapproval, including the protocol, amendments, and informed consent, were reviewed and approved by the national, regional, or investigational center Ethics Committee or Institutional Review Board, in accordance with the International Committee on Harmonization of Good Clinical Practice and applicable country-specific requirements, including US 21 CFR 312.3(b) for constitution of independent ethics committees.

The AMBER study (NCT02817633) is a two-part, multicenter, open-label, phase I study assessing cobolimab as monotherapy or in combination with other drugs in patients with advanced solid tumors. Part 1 included dose escalation/de-escalation, with part 1c assessing patients who received cobolimab in combination with dostarlimab, including patients with locally advanced or metastatic melanoma (Supplementary Fig. S1). In part 1e, cobolimab plus dostarlimab was evaluated in exploratory cohorts of patients who had not received prior anti–PD-(L)1 treatment, including those with locally advanced or metastatic melanoma. Part 2 of AMBER is aiming to assess dose expansion cohorts; in part 2A, dose expansion of cobolimab in patients who received cobolimab in combination with dostarlimab is being assessed in patients with locally advanced or metastatic melanoma who had progressed on prior anti–PD-(L)1 therapy.

The present analysis includes a subpopulation of patients with locally advanced or metastatic melanoma treated with cobolimab plus dostarlimab from parts 1c and 1e who were anti–PD-(L)1–naïve or anti–CTLA-4–naïve and those from part 2A who progressed on prior anti–PD-(L)1 therapy. Enrollment of the first patient into the AMBER study was in July 2016, and the data cutoffs for the present analyses were May 2021 for part 1c, March 2023 for part 1e, and February 2023 for part 2A.

### Eligibility

Patient eligibility was assessed based on compliance with inclusion and exclusion criteria, which are provided in the supplementary text for parts 1c, 1e, and 2A. Briefly, patients ages ≥18 years with a locally advanced or metastatic solid tumor, an Eastern Cooperative Oncology Group (ECOG) performance status from 0 to 1, and adequate organ function were eligible for the AMBER study. For parts 1c and 1e, eligible patients had locally advanced or metastatic melanoma, and for part 1e, those who had not been previously treated with anti–PD-(L)1 or anti–CTLA-4 therapies (may be treatment-naïve) were eligible; prior treatment with BRAF-targeting therapies and patients with previously treated brain metastases were permitted. For part 2A, eligible patients had histologically proven locally advanced (unresectable) or metastatic melanoma that was measurable by CT or magnetic resonance imaging (MRI) per Response Evaluation Criteria in Solid Tumours (RECIST) v1.1 criteria and had progressed following treatment with an anti–PD-(L)1 antibody.

For part 1c, patients were excluded if they had received anti–CTLA-4 treatment within 3 weeks prior to the initiation of study treatment and/or prior treatment with an anti–PD-(L)1, anti–PD-(L)2, anti–TIM-3, or anti–LAG-3, as well as docetaxel, pemetrexed, cisplatin, or carboplatin, agent that resulted in permanent discontinuation due to an AE. In part 1e, patients previously treated with anti-PD-(L)1, anti-TIM-3, or anti-LAG-3 therapy and those with uveal melanoma were excluded. For part 2A, patients were excluded if they had undergone prior treatment with anti–PD-(L)1 or anti–PD-(L)2 therapy that resulted in permanent discontinuation due to an AE and/or had prior treatment with anti–LAG-3 or anti–TIM-3 therapy.

### Treatments

Subsequent to a 21-day screening process (35 days for part 1e), eligible patients received cobolimab 100 mg (1c and 2A only), 300 mg, or 900 mg (1c and 1e only) intravenously plus dostarlimab 500 mg intravenously every 3 weeks. Treatment continued until disease progression, unacceptable toxicity, withdrawal of consent, investigator’s decision, or death (whichever occurred sooner).

### Study endpoints for parts 1c, 1e, and 2A

Primary safety and tolerability endpoints for parts 1c and 1e included safety, tolerability, and the cobolimab recommended phase II dose (RP2D; based on evidence from part 1 of the AMBER study). For parts 1e and 2A, the primary efficacy endpoint was the ORR, defined as the proportion of patients who achieved a complete response or partial response, as assessed by the investigator per RECIST v1.1 criteria. In part 1c, the ORR per RECIST v1.1 criteria was assessed as a secondary endpoint. For parts 1c, 1e, and 2A, the disease control rate (DCR; defined as the percentage of patients who achieved complete response, partial response, or stable disease for a minimum of 16 weeks, as assessed by the investigator per RECIST v1.1) was a secondary endpoint. In part 2A, additional secondary endpoints included progression-free survival (PFS; defined as the time from the first dose to the earlier date of assessment of progression or death by any cause in the absence of progression), overall survival (OS; defined as the time from the date of the first dose of study treatment to the date of death by any cause), and immune-related (ir)ORR and irDCR, as assessed by the investigator per irRECIST. For parts 1c and 1e, irORR and irDCR were exploratory endpoints.

Changes to patient target lesion size from baseline were also assessed. Target lesion size was calculated as the sum of the longest (non-nodal) dimension and shortest (nodal) axes for all target lesions. Patients with no tumor assessments after screening were excluded from this analysis.

### *Post hoc* analyses

The subsets of patients with locally advanced or metastatic melanoma who had not been previously treated with anti–PD-(L)1 or anti–CTLA-4 therapies from parts 1c and 1e, respectively, were combined as part of a *post hoc* integrated analysis. Changes in patient baseline levels of lactate dehydrogenase were assessed *post hoc* for parts 1c/e and 2.

#### *Post hoc* translational analyses: antagonist activity of anti–TIM-3 antibodies on activated human CD4^+^ T cells

Peripheral blood mononuclear cells were separated from healthy donors by Ficoll-Histopaque (Sigma) density gradient centrifugation. CD4^+^ T cells were purified using CD4^+^ T Cell Isolation Kit II (Miltenyi Biotec) following the manufacturer’s protocol and added to standard 96-well tissue culture plates coated with anti-human CD3ε (clone OKT, 2.5 μg/mL) and anti-human CD28 (clone 28, 2.5 µg/mL). The purity of isolated T cells was greater than 95% as assessed by flow cytometry using a mAb against CD3. CD4^+^ T cells (2 × 10^5^ cells/well) were cultured for 48 hours in complete medium (RPMI 1640, 10% FBS and penicillin–streptomycin) and plated in the absence or presence of increasing concentrations of indicated anti–TIM-3 antibodies or an isotype control. After 48 hours, the cell supernatants were harvested, and IL-2 production was assessed by ELISA (R&D Systems, DuoSet) according to the manufacturer’s instructions.

#### *Post hoc* translational analyses: activity of anti–TIM-3 antibodies in the human mixed lymphocyte reaction

Functional antagonist activity of cobolimab was tested in a human CD4^+^ T-cell mixed lymphocyte reaction assay. The mixed lymphocyte reaction assay was carried out using primary human CD4^+^ T cells as responders and DCs as stimulators that were derived from human monocytes of a different donor after 7 days of culture with granulocyte-macrophage colony-stimulating factor (GM-CSF) and IL-4. DCs and CD4^+^ T cells were incubated in the presence or absence of cobolimab antibodies for 48 hours, and then culture supernatants were harvested with IL-2 levels quantified by ELISA (R&D Systems, DuoSet) to determine T-cell activation as measured by the level of IL-2 secreted.

#### *Post hoc* translational analyses: single-cell RNA sequencing expression data in anti–PD-1 nonresponders

**Figure 1.**
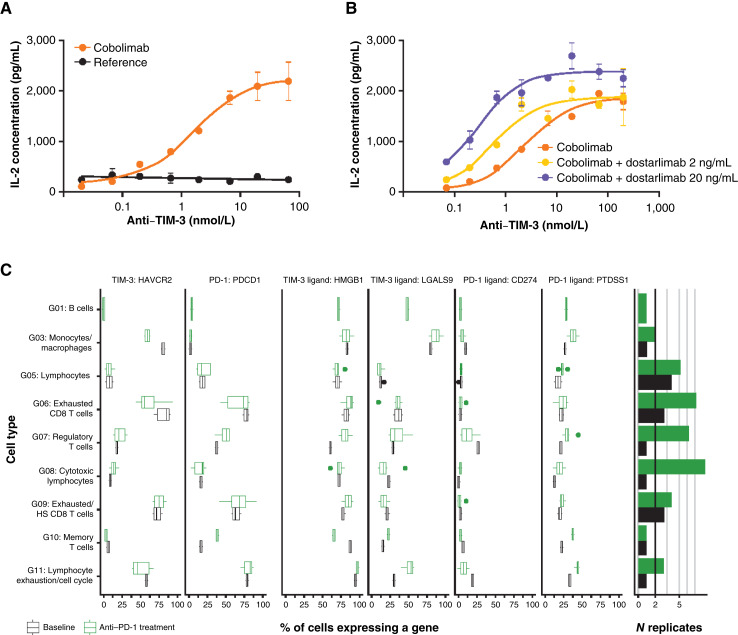
CD4^+^ T cells induced by cobolimab (**A**) and combined cobolimab and dostarlimab (**B**). Percentage of single cells with transcript expression of TIM-3, PD-1, and their ligands in baseline and anti–PD-1–treated samples across key immune cells (**C**). Note: In **C**, observations shown for >50 cells; only nonresponder samples were included in the analysis. IL, interleukin; PD-1, programmed death-1; TIM-3, T-cell immunoglobulin and mucin-domain containing 3.

To determine cell types with PD-1, TIM-3, and TIM-3 or PD-1 ligand expression, single-cell RNA sequencing data from Sade-Feldman and colleagues (31) were analyzed, with cell type annotations provided by the authors. Samples were sorted for CD45^+^ cells prior to generating single-cell transcriptomics data. The percentage of cells with >0 counts for genes of interest in each cell type were then assessed with a single percentage obtained per patient sample (replicate). Samples were filtered for anti–PD-1 nonresponders, as annotated by the publication. Data presented in Fig. 1C were also filtered for data points with >50 cells per sample and cell type.

### Assessments and safety

To assess the extent of the disease, CT or MRI evaluations were conducted every 8 weeks (56 ± 7 days) from the date of the first dose of study treatment. For patients who remained on treatment after 1 year, imaging was performed every 12 weeks (84 ± 7 days). Radiographic tumor assessments were performed independent of dose delays and/or interruptions and/or at any time disease progression was suspected. Following disease progression or treatment discontinuation, patients had safety follow-up visits 30 (±7) and 90 (±14) days after the last dose of study treatment, unless they discontinued the study.

For safety assessments, AEs were coded using the Medical Dictionary for Regulatory Activities (MedDRA) v24.0 for part 1c and v25.1 for parts 1e and 2A, assessed by investigators according to Common Terminology Criteria for Adverse Events (CTCAE) v4.03. AEs were recorded by frequency and severity and included treatment-emergent AEs (TEAE), TRAEs, and irTEAEs. Information on AEs was collected and recorded from the day of signed informed consent until 90 days after treatment or until initiation of alternative anticancer therapy, whichever occurred first.

### Statistical analysis

Efficacy and safety analyses were performed for the safety population, defined as patients who received any amount of cobolimab.

For part 2A, a sample size of approximately 88 patients (44 per treatment arm) was planned in accordance with a Simons’ two-stage minimax design, with the final sample size for each dose level determined following the ongoing examination of the totality of the data observed, including receptor occupancy, safety, and efficacy data. The minimax design assumed a null hypothesis of a 5% true response rate tested against a one-sided alternative when the true response rate is 15% (one-sided type I error rate: 10%; power: 80%).

Categorical variables were summarized as numbers and percentages, and continuous variables were summarized as median, first and third quartiles, and minimum and maximum values. For the response rates, point estimates and Clopper–Pearson two-sided 95% confidence intervals (CI) were calculated (32). Time-to-event analyses for part 2A were performed using Kaplan–Meier methods, with 95% CIs calculated using the Brookmeyer and Crowley method (33).

### RRID resources

R Project for Statistical Computing (RRID: SCR_001905); cowplot (RRID: SCR_018081); tidyverse (RRID: SCR_019186); dplyr (RRID: SCR_016708); tidyr (RRID: SCR_017102), ggplot2 (RRID: SCR_014601); purrr (RRID: SCR_021267); knitr (RRID: SCR_018533); and BbrowserX (RRID: SCR_025984; utilized by BioTuring).

### Data availability

Please refer to https://www.gsk-studyregister.com/en/ to access GSK’s data sharing policies and as applicable seek anonymized subject-level data via https://vivli.org/ourmember/gsk/. To access data for other types of GSK sponsored research, for study documents without patient-level data, and for clinical studies not listed, please send an email to ww.share-support@gsk.com.

## Results

### Preclinical characterization of cobolimab

Preclinical data contributed to the rationale to study combined PD-1 and TIM-3 blockade in patients with advanced melanoma. In biochemical and cellular assays, cobolimab had high affinity for human and cynomolgus monkey TIM-3 but did not bind appreciably to murine TIM-3 (Supplementary Fig. S2; Supplementary Table S1). *In vitro* and *ex vivo* assays demonstrated that cobolimab increased T-cell activation as a single agent (Fig. 1A) or in combination with dostarlimab (Fig. 1B), respectively, via increased IL-2 production compared with a human IgG4 isotype reference antibody in a concentration-dependent manner. Furthermore, a reanalysis of external single-cell RNA sequencing of patients who were nonresponders to anti–PD-1 therapy and with available baseline and treated samples showed that observed TIM-3 expression (*HAVCR2*) was moderately decreased following anti–PD-1 therapy (Fig. 1C).

### Study population

For the current integrated analysis, 28 patients who received treatment in parts 1c and 1e [cobolimab 100 mg: *n* = 3 (1c); 300 mg: *n* = 14 (1c: *n* = 4; 1e: *n* = 10); and 900 mg: *n* = 11 (1c: *n* = 3; 1e: *n* = 8)] and 43 patients who received treatment in part 2A (100 mg: *n* = 14; 300 mg: *n* = 29) were included. Patient baseline demographics and characteristics are summarized in Table 1. In brief, the median patient age was 68.0 years (range, 39–87) in parts 1c/1e and 65.0 years (range, 30–90) in part 2A. For parts 1c/1e and 2A, the most common primary tumor site was the skin [23 (82.1%) and 34 (79.1%), respectively], and 3 (10.7%) and 2 (4.7%) patients had uveal melanoma, respectively. Baseline lactate dehydrogenase levels were above the upper limit of normal in 14 (50.0%) patients in parts 1c/1e and 24 (55.8%) patients in part 2A. In parts 1c/1e, most patients (71.4%) had no lines of prior treatment, whereas in part 2A, 90.7% of patients had received ≥2 lines of prior treatment. Representativeness of study patients compared with the wider patient population is shown in Supplementary Table S2.

**Table 1.** Baseline patient demographics and clinical characteristics.

Characteristic	Parts 1c and 1e				Part 2A		
	Cobolimab 100 mg + dostarlimab (*n* = 3^a^)	Cobolimab 300 mg + dostarlimab (*n* = 14^b^)	Cobolimab 900 mg + dostarlimab (*n* = 11^c^)	Total (*N* = 28)	Cobolimab 100 mg + dostarlimab (*n* = 14)	Cobolimab 300 mg + dostarlimab (*n* = 29)	Total (*N* = 43)
**Age, median (range), years**	D^d^	70.0 (48–87)	66.0 (39–74)	68.0 (39–87)	63.5 (43–74)	65.0 (30–90)	65.0 (30–90)
**Sex, *n* (%)**							
Female	D^d^	D^d^	D^d^	12 (42.9)	D^d^	D^d^	18 (41.9)
Male	D^d^	D^d^	D^d^	16 (57.1)	D^d^	D^d^	25 (58.1)
**Race, *n* (%)**							
White	D^d^	D^d^	D^d^	26 (92.9)	13 (92.9)	27 (93.1)	40 (93.0)
Deidentified	D^d^	D^d^	D^d^	2 (7.1)	D^d^	D^d^	3 (7.0)
**Ethnicity, *n* (%)**							
Not Hispanic or Latino	D^d^	D^d^	D^d^	23 (82.1)	14 (100)	24 (82.8)	38 (88.4)
Deidentified	D^d^	D^d^	D^d^	5 (17.9)	D^d^	D^d^	5 (11.6)
**ECOG PS, *n* (%)**							
0	1 (33.3)	11 (78.6)	7 (63.6)	19 (67.9)	6 (42.9)	13 (44.8)	19 (44.2)
1	2 (66.7)	3 (21.4)	4 (36.4)	9 (32.1)	8 (57.1)	16 (55.2)	24 (55.8)
**Primary tumor site, *n* (%)**							
Skin	3 (100)	13 (92.9)	7 (63.6)	23 (82.1)	9 (64.3)	25 (86.2)	34 (79.1)
Anorectal mucosal	0	0	1 (9.1)	1 (3.6)	3 (21.4)	3 (10.3)	6 (14.0)
Uveal	0	0	3 (27.3)	3 (10.7)	2 (14.3)	0	2 (4.7)
Other–nasal cavity	0	0	0	0	0	1 (3.4)	1 (2.3)
Unknown	0	1 (7.1)	0	1 (3.6)	0	0	0
**Prior lines of treatment^e^, *n* (%)**							
0	2 (66.7)	11 (78.6)	7 (63.6)	20 (71.4)	0	0	0
1	1 (33.3)	3 (21.4)	4 (36.4)	8 (28.6)	2 (14.3)	2 (6.9)	4 (9.3)
≥2	0	0	0	0	12 (85.7)	27 (93.1)	39 (90.7)
**PD-L1 status^f^, *n* (%)**							
TPS ≥50%	0	2 (14.3)	1 (9.1)	3 (10.7)	0	0	0
TPS 1%–49%	0	1 (7.1)	0	1 (3.6)	0	0	0
TPS <1%	0	0	2 (18.2)	2 (7.1)	0	0	0
Missing	3 (100)	11 (78.6)	8 (72.7)	22 (78.6)	14 (100)	29 (100)	43 (100)
**Known genetic abnormality, *n* (%)**							
*BRAF* (other)	0	2 (14.3)	0	2 (7.1)	1 (7.1)	8 (27.6)	9 (20.9)
*BRAF* (*V600E*)	0	1 (7.1)	1 (9.1)	2 (7.1)	6 (42.9)	6 (20.7)	12 (27.9)
*GNAQ*	n/a	n/a	n/a	n/a	1 (7.1)	0	1 (2.3)
*NRAS*	0	1 (7.1)	1 (9.1)	2 (7.1)	1 (7.1)	4 (13.8)	5 (11.6)
Other	0	0	1 (9.1)	1 (3.6)	0	2 (6.9)	2 (4.7)
Missing	3 (100)	10 (71.4)	8 (72.7)	21 (75.0)	5 (35.7)	9 (31.0)	14 (32.6)
**Baseline LDH levels, *n* (%)**							
≤ULN	1 (33.3)	10 (71.4)	3 (27.3)	14 (50.0)	3 (21.4)	16 (55.2)	19 (44.2)
>ULN	2 (66.7)	4 (28.6)	8 (72.7)	14 (50.0)	11 (78.6)	13 (44.8)	24 (55.8)

Abbreviations: BRAF, v-raf murine sarcoma viral oncogene homolog B1; D, deidentified; ECOG PS, Eastern Cooperative Oncology Group performance status; GNAQ, G protein subunit α Q; LDH, lactate dehydrogenase; NRAS, neuroblastoma RAS viral oncogene homolog; PD-L1, programmed death ligand 1; TPS, tumor proportion score; ULN, upper limit of normal.

a Three patients from part 1c.

b Four patients from part 1c and 10 patients from part 1e.

c Three patients from part 1c and eight patients from part 1e.

d Data deidentified for demographic variables if at least one treatment arm had an *n* < 11.

e Prior treatments in parts 1c/1e were binimetinib, cobimetinib, dabrafenib, encorafenib, interferon therapy (IFN), IFN α-2b, XL888, other anti-neoplastic agents, pegylated IFN α-2b, trametinib, ulixertinib, and vemurafenib, each reported in one patient, and the main prior treatments in part 2 were pembrolizumab, ipilimumab, and nivolumab, reported in >40% of the total population.

f Tested locally.

### Safety

TEAEs are summarized in Table 2. TEAEs occurred in all 28 patients included in parts 1c/1e and 40 (93.0%) patients included in part 2A, with a similar incidence across cobolimab dose cohorts. TRAEs occurred in 25 (89.3%) patients in parts 1c/1e and 26 (60.5%) patients in part 2A. Grade ≥3 TRAEs were experienced by 8 (28.6%) patients in parts 1c/1e and 5 (11.6%) patients in part 2A; there were no deaths due to TRAEs.

**Table 2.** Overview of safety results.

AE^a^, *n* (%)	Parts 1c and 1e				Part 2A		
	Cobolimab 100 mg + dostarlimab (*n* = 3)	Cobolimab 300 mg + dostarlimab (*n* = 14)	Cobolimab 900 mg + dostarlimab (*n* = 11)	Total (*N* = 28)	Cobolimab 100 mg + dostarlimab (*n* = 14)	Cobolimab 300 mg + dostarlimab (*n* = 29)	Total (*N* = 43)
**TEAEs**	3 (100)	14 (100)	11 (100)	28 (100)	13 (92.9)	27 (93.1)	40 (93.0)
**TRAEs**	2 (66.7)	14 (100)	9 (81.8)	25 (89.3)	8 (57.1)	18 (62.1)	26 (60.5)
**Grade ≥3 TEAEs**	3 (100)	7 (50.0)	6 (54.5)	16 (57.1)	5 (35.7)	11 (37.9)	16 (37.2)
**Grade ≥3 TRAEs**	1 (33.3)	2 (14.3)	5 (45.5)	8 (28.6)	1 (7.1)	4 (13.8)	5 (11.6)
**TESAEs^b^**	3 (100)	7 (50.0)	4 (36.4)	14 (50.0)	4 (28.6)	7 (24.1)	11 (25.6)
**TRSAEs**	0	2 (14.3)	2 (18.2)	4 (14.3)	1 (7.1)	3 (10.3)	4 (9.3)
**irTEAEs**	N/A^c^	N/A^c^	N/A^c^	N/A^c^	2 (14.3)	4 (13.8)	6 (14.0)
**irTESAEs**	N/A^c^	N/A^c^	N/A^c^	N/A^c^	1 (7.1)	2 (6.9)	3 (7.0)
Rash	N/A^c^	N/A^c^	N/A^c^	N/A^c^	0	1 (3.4)	1 (2.3)
Myositis	N/A^c^	N/A^c^	N/A^c^	N/A^c^	0	1 (3.4)	1 (2.3)
Hypophysitis	N/A^c^	N/A^c^	N/A^c^	N/A^c^	1 (7.1)	0	1 (2.3)
**TEAEs leading to study treatment discontinuation**	0	3 (21.4)	1 (9.1)	4 (14.3)	0	1 (3.4)	1 (2.3)
**TEAEs leading to study treatment delay**	1 (33.3)	3 (21.4)	1 (9.1)	5 (17.9)	0	2 (6.9)	2 (4.7)
**Fatal TEAEs**	1 (33.3)	0	0	1 (3.6)	0	0	0
**Fatal TRAEs**	0	0	0	0	0	0	0

Abbreviations: ir, immune-related; TEAE, treatment-emergent adverse event; TESAE, treatment-emergent serious adverse event; TRAE, treatment-related adverse event; TRSAE, treatment-related serious adverse event.

a AEs were coded using the Medical Dictionary for Regulatory Activities v24.0 for part 1c and v25.1 for parts 1e and 2A.

b Most common TESAE (*n* ≥ 2) was constipation (part 2A, 100 mg, *n* = 2).

c Data not available.

In parts 1c/1e and 2A, treatment-emergent serious AEs (TESAE) were experienced by 14 (50.0%) and 11 (25.6%) patients, respectively, the most common of which were acute kidney injury for 2 (7.1%) patients in parts 1c/1e and constipation for 2 (4.7%) patients in part 2A.

In part 2A, TEAEs and irTESAEs were reported in 6 (14.0%) and 3 (7.0%) patients, respectively. irTESAEs included rash, myositis, and hypophysitis in 1 (2.3%) patient each (Table 2). irTEAEs and irTESAEs were not reported in parts 1c/1e.

### Efficacy

**Figure 2.**
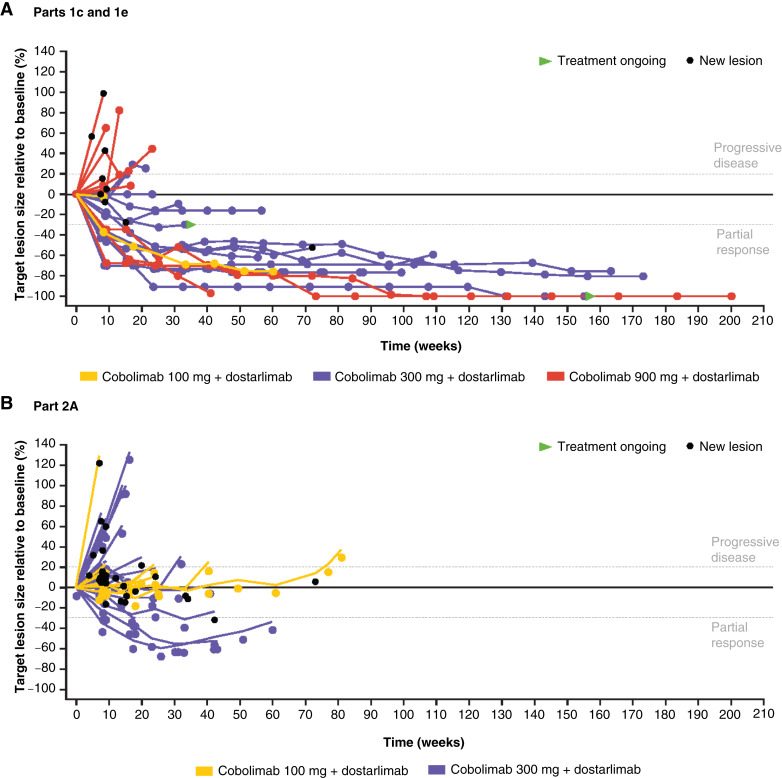
Relative change in target lesion size across cobolimab dose ranges in parts 1c and 1e (*N* = 27; **A**) and part 2A (*N* = 39; **B**). Note: *N* number is defined as the safety population minus one patient from parts 1c/e and four patients from part 2A with target lesion size not available postbaseline, including those with no postbaseline tumor assessments.

The response data, assessed using RECIST v1.1 and irRECIST, are summarized in Table 3. Across all cobolimab dose levels, the confirmed ORR was 42.9% (95% CI, 24.5–62.8) in parts 1c/1e and 4.7% (95% CI, 0.6–15.8) in part 2A. The DCR was 53.6% (95% CI, 33.9–72.5) in parts 1c/1e and 20.9% (95% CI, 10.0–36.0) in part 2A. The ORR across the cobolimab dose levels in parts 1c/1e and 2A ranged from 27.3% to 57.1% and 0% to 6.9%, and the DCR ranged from 33.3% to 71.4% and 17.2% to 28.6%, respectively. The highest ORRs [parts 1c/1e: 57.1% (95% CI, 28.9–82.3); part 2A: 6.9% (95% CI, 0.8–22.8)] were observed in the cobolimab 300 mg cohorts (parts 1c/1e, *n* = 14; part 2A, *n* = 29), and the highest DCR was observed in the cobolimab 300 mg cohort for parts 1c/1e [71.4% (95% CI, 41.9–91.6)] and the cobolimab 100 mg cohort for part 2A [28.6% (95% CI, 8.4–58.1); *n* = 14]. Across cobolimab cohorts, the irORR was the same as the ORR. The irDCR was 57.1% (16/28; 95% CI, 37.2–75.5) in parts 1c/1e and 25.6% (11/43; 95% CI, 13.5–41.2) in part 2A. Change in target lesion size relative to baseline for parts 1c/1e and 2A is shown in Fig. 2.

**Table 3.** ORR, DCR, and BOR in patients treated with cobolimab 100, 300, or 900 mg plus dostarlimab.

Response, *n* (%)	Parts 1c and 1e				Part 2A		
	Cobolimab 100 mg + dostarlimab (*n* = 3)	Cobolimab 300 mg + dostarlimab (*n* = 14)	Cobolimab 900 mg + dostarlimab (*n* = 11)	Total (*N* = 28)	Cobolimab 100 mg + dostarlimab (*n* = 14)	Cobolimab 300 mg + dostarlimab (*n* = 29)	Total (*N* = 43)
**ORR**	1 (33.3)	8 (57.1)	3 (27.3)	12 (42.9)	0	2 (6.9)	2 (4.7)
(95% CI)	(0.8–90.6)	(28.9–82.3)	(6.0–61.0)	(24.5–62.8)		(0.8–22.8)	(0.6–15.8)
**DCR**	1 (33.3)	10 (71.4)	4 (36.4)	15 (53.6)	4 (28.6)	5 (17.2)	9 (20.9)
(95% CI)	(0.8–90.6)	(41.9–91.6)	(10.9–69.2)	(33.9–72.5)	(8.4–58.1)	(5.8–35.8)	(10.0–36.0)
**BOR per RECIST v1.1**							
CR	0	0	0	0	0	0	0
PR	1 (33.3)	8 (57.1)	3 (27.3)	12 (42.9)	0	2 (6.9)	2 (4.7)
SD^a^	0	2 (14.3)	1 (9.1)	3 (10.7)	4 (28.6)	3 (10.3)	7 (16.3)
Progressive disease	0	4 (28.6)	7 (63.6)	11 (39.3)	8 (57.1)	22 (75.9)	30 (69.8)
Not evaluable	1 (33.3)	0	0	1 (3.6)	0	0	0
Not done^b^	1 (33.3)	0	0	1 (3.6)	2 (14.3)	2 (6.9)	4 (9.3)
**irORR**	1 (33.3)	8 (57.1)	3 (27.3)	12 (42.9)	0	2 (6.9)	2 (4.7)
(95% CI)	(0.8–90.6)	(28.9–82.3)	(6.0–61.0)	(24.5–62.8)		(0.8–22.8)	(0.6–15.8)
**irDCR**	1 (33.3)	11 (78.6)	4 (36.4)	16 (57.1)	4 (28.6)	7 (24.1)	11 (25.6)
(95% CI)	(0.8–90.6)	(49.2–95.3)	(10.9–69.2)	(37.2–75.5)	(8.4–58.1)	(10.3–43.5)	(13.5–41.2)
**BOR per irRECIST**							
irCR	0	0	1 (9.1)	1 (3.6)	0	0	0
irPR	1 (33.3)	8 (57.1)	2 (18.2)	11 (39.3)	0	2 (6.9)	2 (4.7)
irSD^a^	0	3 (21.4)	1 (9.1)	4 (14.3)	4 (28.6)	5 (17.2)	9 (20.9)
irPD	0	3 (21.4)	6 (54.5)	9 (32.1)	8 (57.1)	18 (62.1)	26 (60.5)
Not evaluable	1 (33.3)	0	1 (9.1)	2 (7.1)	0	2 (6.9)	2 (4.7)
Not done^b^	1 (33.3)	0	0	1 (3.6)	2 (14.3)	2 (6.9)	4 (9.3)

Abbreviations: BOR, best overall response; CI, confidence interval; CR, complete response; DCR, disease control rate; ir, immune-related; ORR, overall response rate; PD, progressive disease; PR, partial response; RECIST v1.1, Response Evaluation Criteria in Solid Tumours version 1.1; SD, stable disease.

a SD/irSD confirmed with a minimum of 16-week duration.

b Patients in the safety population who had no postbaseline tumor assessments.

**Figure 3.**
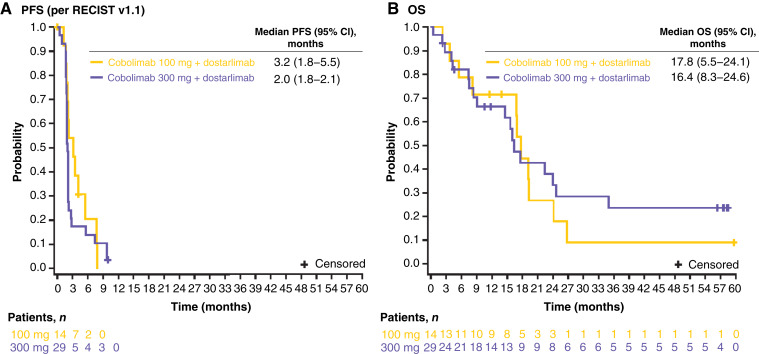
Kaplan–Meier plot of PFS (**A**) and OS (**B**) in all patients in part 2A. CI, confidence interval; OS, overall survival; PFS, progression‐free survival; RECIST v1.1, Response Evaluation Criteria for Solid Tumours, version 1.1.

The PFS and OS of all patients in part 2A are shown in Fig. 3. The median PFS in the part 2A population was 3.2 (95% CI, 1.8–5.5) and 2.0 (95% CI, 1.8–2.1) months for patients who received 100 and 300 mg of cobolimab (plus dostarlimab), respectively. The median OS was 17.8 (95% CI, 5.5–24.1) months in the 100 mg cohort and 16.4 (95% CI, 8.3–24.6) months in the 300 mg cohort.

## Discussion

In AMBER parts 1c/1e and 2A, the safety and efficacy of the combination of cobolimab plus dostarlimab across the cobolimab 100, 300, and 900 mg doses were assessed in patients with locally advanced or metastatic melanoma, including subsets of patients who were immunotherapy-naïve and those who had progressed on prior anti–PD-(L)1 therapy.

Across the cohorts, cobolimab in combination with dostarlimab was well tolerated, with a low incidence of grade ≥3 TRAEs. This finding is in line with previous studies of anti–PD-(L)1 combinations; a similar incidence of TRAEs was reported for combined nivolumab (anti–PD-1) and relatlimab (anti–LAG-3) treatment for patients with advanced melanoma who are treatment-naïve (RELATIVITY-047) and PD-(L)1–experienced (RELATIVITY-020; refs. 4, 34). The manageable safety profile of cobolimab and dostarlimab has also been demonstrated in patients with locally advanced or metastatic NSCLC in the AMBER study (35). Collectively, these data highlight the relative tolerability of a combined cobolimab and dostarlimab treatment option for patients with solid tumors.

The preclinical data showed that combined cobolimab and dostarlimab enhanced T-cell activation and reduced TIM-3 expression in key immune cells for patients with prior ICI treatment. This is consistent with the observed efficacy described here in which a lower ORR was observed for patients previously treated with anti–PD-(L)1 (part 2A) versus anti–PD-(L)1–naïve patients (parts 1c/1e *post hoc* analysis).

For patients in parts 1c/1e, compared with the observed ORRs in the cobolimab 100 mg (33.3%) or cobolimab 900 mg (27.3%) cohorts, the ORR was greatest in the cobolimab 300 mg (57.1%) cohort. It is important to note the limited sample size in this cohort and the inclusion of three patients with uveal melanoma in the 900 mg cohort; however, a similar trend was observed for patients with anti–PD-1 relapsed or refractory disease in part 2A, wherein the ORR of the cobolimab 300 mg cohort was greater (6.9%) than that of the cobolimab 100 mg cohort (0%). Given the safety findings reported here and the pharmacokinetic, pharmacodynamic, receptor occupancy, efficacy, and safety data from the other patient cohorts in the AMBER study (29, 35, 36), the cobolimab 300 mg dose level was selected as the RP2D.

The observed ORRs in this study for patients with melanoma who were PD-1–naïve (42.9%) or relapsed or refractory (4.7%) are in line with previous studies examining these patient settings [43.1% (RELATIVITY-047) for PD-1–naïve and 9.2% to 12.0%, (RELATIVITY-020) for relapsed or refractory; refs. 37, 38]. In combination, these findings contribute to the body of work underscoring the importance of evaluating anti–TIM-3 inhibitors in the immunotherapy-naïve setting.

Although indirect comparisons to other studies are speculative in nature and should be considered cautiously, particularly due to the small sample size in this cohort, the ORR for the cobolimab 300 mg cohort (57.1%) compares favorably with reported ORRs for PD-1 monotherapy in a PD-1–naïve setting (∼39–45%; refs. 5, 39).

Likewise, this value is in line with prior studies assessing combination therapies for patients with PD-1–naïve melanoma, including PD-1 nivolumab combined with LAG-3 inhibitor relatlimab (43.1%, RELATIVITY-047) and nivolumab combined with CTLA-4 inhibitor ipilimumab (57.6%, CheckMate-067; refs. 4, 5). Furthermore, this finding aligns with results from a phase II study assessing cobolimab plus dostarlimab treatment in patients with advanced hepatocellular carcinoma, which demonstrated encouraging interim results with a reported ORR of 46% (40), supporting the potential for therapeutic benefit in other cancers as well in the immunotherapy-naïve setting.

The current translational data analysis, which suggested that patients with melanoma whose disease had progressed on prior ICI treatment had reduced observed TIM-3 levels, contributes to emerging evidence that, due to adaptive resistance, expression of TIM-3 is upregulated in anti–PD-(L)1–resistant tumors (41–43). In combination with the reported efficacy, this suggests that TIM-3 blockade may reverse T-cell exhaustion and indicates the potential synergistic effect of concurrently targeting TIM-3 and PD-(L)1 in patients with melanoma who are immunotherapy-naïve, particularly before anti–PD-(L)1 resistance develops, i.e., in the first-line setting. Well-powered, randomized studies with suitable controls will be helpful to assess the full potential of such combinations.

This exploratory study further supports the rationale for dual TIM-3 and PD-(L)1 blockade in advanced or metastatic solid tumors, particularly in the immunotherapy-naïve setting. These data serve as a foundation for further evaluation of cobolimab in multiple indications, including anti–PD-(L)1 refractory and relapsed advanced NSCLC (COSTAR Lung, NCT04655976), PD-1–naïve hepatocellular carcinoma (NCT03680508), and high-risk resectable melanoma (Neo-MEL-T, NCT04139902). A key strength of the AMBER study was that three dose levels of cobolimab were explored (100, 300, 900 mg) in combination with dostarlimab to find the nominal dose for further exploration, with an intermediate dose level being picked as RP2D based on aggregate clinical and translational data. Limitations of this study include the lack of a dostarlimab control arm, which precluded any definitive conclusion about the contribution of TIM-3 blockade to the observed activity in PD-(L)1–naïve melanoma. In addition, patient numbers in the current analysis, including the preclinical translational analysis, were relatively small, limiting interpretation and the ability to compare results with other studies.

In conclusion, for patients with advanced metastatic melanoma in the AMBER study, cobolimab plus dostarlimab had a manageable safety profile and demonstrated preliminary antitumor activity, particularly in patients naïve to anti–PD-(L)1 and CTLA-4 therapy. These results suggest that targeting TIM-3 and PD-1 as a combination therapy in melanoma may offer an alternative therapeutic approach and merits further investigation for patients with advanced solid tumors.

## Supplementary Material

Supplementary Text1 Supplementary inclusion/exclusion criteria

Supplementary Figure S1 AMBER Part 1c/1e and Part 2A design

Supplementary Figure S2 Cobolimab binding to native TIM-3 in T cells and monocytes

Supplementary Table S1 Cobolimab binding affinity

Supplementary Table S2 Representativeness of study patients
