# Supplementary material for: Combined Targeting of PD-1 and TIM-3 in Patients with Locally Advanced or Metastatic Melanoma: AMBER Cohorts 1c, 1e, and 2A
Source: Clin Cancer Res. 2025 Jun 24;31(16):3433–42. doi: 10.1158/1078-0432.CCR-25-0884 (PMC12351273; doi:10.1158/1078-0432.CCR-25-0884)
Supplement: Supplementary Text1 — Supplementary inclusion/exclusion criteria [file ccr-25-0884_supplementary_text1_suppts1.docx]

### Supplementary Text

**Full inclusion and exclusion criteria**

**Inclusion criteria for Parts 1c, 1e, and 2A**

- Patient were at least 18 years of age
- Patients with locally advanced or metastatic solid tumor who met the following requirements:
  - Part 1: Disease progression after treatment with available therapies known to confer clinical benefit or who were intolerant to treatment (except for Part 1e, see below)
    - Part 1e: Patients with locally advanced or metastatic melanoma who had not been previously treated with anti-PD-(L)1, or anti-CTLA-4 therapies. Patient may have been treatment-naïve
  - Part 2: Histologically proven locally advanced (unresectable) or metastatic melanoma that was measurable by CT or MRI per RECIST v1.1 criteria and had progressed following treatment with an anti-PD-(L)1 antibody
- Biopsies
  - Part 1: Patients must have had archival tumor tissue available that was formalin-fixed and paraffin-embedded. Tumor tissue must have been requested from off-site locations and confirmed available prior to dosing
    - For patients in Part 1 who did not have archival tissue, new biopsies would need to have been performed
    - For patients enrolled in Part 1 who agreed to optional serial biopsies, biopsies must be obtained prior to treatment, approximately 4 to 6 weeks after treatment and, if possible, at the time of disease progression (EOT Visit)
  - Part 2A: Required to have had fresh tumor tissue biopsy prior to dosing. Archival tissue could have also been provided (if available) to enable longitudinal analysis of tumor biomarkers
    - For the archival sample, tumor tissue must have been requested from offsite locations and confirmed available prior to dosing
    - If a patient had a biopsy prior to entering the 21-day screening period and within approximately 12 weeks of study treatment, that biopsy may have been accepted as the baseline fresh biopsy
    - All patients were required to have lesions amenable to biopsy and to agree to tumor biopsies prior to the initiation of treatment (as noted above) approximately 4 to 6 weeks after initiating treatment, and, if possible, upon treatment discontinuation (for patients with progressive disease)
- Female patients of childbearing potential must have had a negative serum or urine pregnancy test within 72 hours prior to the date of the first dose of study medication or be of nonchildbearing potential. Non-childbearing potential was defined as:
  - ≥45 years of age and no menses for >1 year
  - Amenorrhoeic for <2 years without a hysterectomy and oophorectomy and a follicle stimulating hormone value in the postmenopausal range upon pre-study (screening) evaluation.
  - Post hysterectomy, bilateral oophorectomy, or tubal ligation. Documented hysterectomy or oophorectomy must be confirmed with medical records of the actual procedure or confirmed by an ultrasound. Tubal ligation must be confirmed with medical records of the actual procedure, otherwise the patient must fulfill the criteria in Inclusion Criteria regarding the solid tumor
- Contraception:
  - Female patients of childbearing potential (see above) must have agreed to use a highly effective method of contraception with their partner starting with the Screening Visit through 150 days after the last dose of study therapy
- Patient had an ECOG performance status of ≤1
- Patient has adequate organ function, defined as:
  - ANC ≥1,500/μL
  - Platelets ≥100,000/μL
  - Hemoglobin ≥9 g/dL or ≥5.6 mmol/L
  - Serum creatinine ≤1.5×ULN or calculated creatinine clearance rate (CCr) ≥50 mL/min using Cockcroft-Gault equation for patients with creatinine levels >1.5× institutional ULN
  - Total bilirubin ≤1.5×ULN (2.0×ULN in patients with Gilbert’s syndrome) AND direct bilirubin ≤1×ULN
  - AST and ALT ≤2.5×ULN unless liver metastases are present, in which case they must be ≤5×ULN
  - International normalized ratio (INR) or prothrombin time (PT) ≤1.5×ULN unless patient is receiving anticoagulant therapy as long as PT or partial thromboplastin time (PTT) is within therapeutic range of intended use of anticoagulants. Activated partial thromboplastin time (aPTT) ≤1.5×ULN unless patient is receiving anticoagulant therapy as long as PT or PTT is within therapeutic range of intended use of anticoagulants.
  - Albumin ≥3.0 g/dL

**Exclusion criteria for Parts 1c, 1e, and 2A**

- Patient has received prior therapy as defined below:
  - Part 1c: Anti-CTLA-4 within 3 weeks (ie, 21 days) prior to initiation of study treatment and/or prior treatment with anti-PD-(L)1, anti-PD-L2, anti-Tim-3, or anti-LAG-3, docetaxel, pemetrexed, cisplatin, or carboplatin agent that resulted in permanent discontinuation due to an AE
  - Part 1e: Prior treatment with an anti-PD-(L)1, anti-Tim-3, or anti-LAG-3; patients with uveal melanoma
  - Part 2 Cohort A:
    - Prior treatment with anti-PD-(L)1, or anti-PD-L2 agent that resulted in permanent discontinuation due to an adverse event
    - Prior treatment with an anti-LAG-3 or anti-Tim-3
- History of Grade ≥3 immune-related AE with prior immunotherapy, with the exception of non-clinically significant lab abnormalities
  - Note (for all patients except Part 1e): Patient may have been considered eligible if anti-PD-(L)1 treatment was tolerated without corticosteroids or other immunosuppressive therapy following a Grade 3 irAE on prior anti-CTLA-4 therapy
- Patient has known uncontrolled central nervous system (CNS) metastases and/or carcinomatous meningitis
  - Note: Patients with previously treated brain metastases may have participated, provided they were stable (without evidence of progression by imaging for at least 4 weeks prior to the first dose of study treatment and any neurologic symptoms have returned to baseline), had no evidence of new or enlarging brain metastases, and were clinically stable off steroids for at least 7 days prior to study treatment. Carcinomatous meningitis precludes a patient from study participation regardless of clinical stability
- Patient had known additional malignancy that progressed or required active treatment within the last 2 years. Patients with a prior or concurrent malignancy whose natural history or treatment did not have the potential to interfere with the safety or efficacy assessment of the investigational regimen may have been included only after discussion with the Medical Monitor
- Patient was considered a poor medical risk due to a serious, uncontrolled medical disorder, nonmalignant systemic disease or active infection requiring systemic therapy. Specific examples include, but are not limited to, active, non-infectious pneumonitis; uncontrolled chronic obstructive pulmonary disease (COPD); uncontrolled ventricular arrhythmia; recent (within 90 days) myocardial infarction; uncontrolled major seizure disorder; unstable spinal cord compression; superior vena cava syndrome; or any psychiatric or substance abuse disorders that would have interfered with cooperation with the requirements of the study (including obtaining informed consent)
- Patient was pregnant or breastfeeding or expecting to conceive children within the projected duration of the study, starting with the Screening Visit through 150 days after the last dose of study treatment
- Patient had a diagnosis of immunodeficiency or was receiving systemic steroid therapy or any other form of immunosuppressive therapy within 7 days prior to the first dose of study treatment
- Patient had a known history of human immunodeficiency virus (HIV) infection or HIV 1/2 antibodies
- Patient had known active hepatitis B (eg, hepatitis B surface antigen [HBsAg] reactive) or hepatitis C (eg, hepatitis C virus ribonucleic acid [HCV RNA] [qualitative] is detected)
- Patient had an active autoimmune disease that required systemic treatment (ie, with use of disease-modifying agents, corticosteroids or immunosuppressive drugs). Replacement therapy (eg, thyroxine, insulin, or physiologic corticosteroid replacement therapy up to prednisone 5 mg or equivalent for adrenal or pituitary insufficiency, etc.) was not considered a form of systemic treatment. Use of inhaled steroids, topical steroids, local injection of steroids, and steroid eye drops was allowed
- Patient has a history of pneumonitis
- Patient had not recovered (ie, to Grade ≤1 or to baseline) from radiation- and chemotherapy-induced AEs, had received transfusion of blood products (including platelets or red blood cells), or had received administration of colony-stimulating factors (including GM-CSF or recombinant erythropoietin) within 3 weeks prior to the first dose of study drug
- Patient was currently participating and receiving study therapy or had participated in a study of an investigational agent and received investigational therapy or used an investigational device within 4 weeks prior to the first dose of study drug.
- Patient had received prior anti-cancer therapy (chemotherapy, targeted therapies, radiotherapy, or immunotherapy) within 21 days, or less than 5 times the half-life of the most recent therapy prior to study Day 1, whichever is shorter. Note: palliative radiation therapy to a small field ≥1 week prior to Day 1 of study treatment may have been allowed
- Patient had not recovered adequately (Grade ≤1) from AEs and/or complications from any major surgery prior to starting therapy
- Patient had received a vaccine other than a vaccine against severe acute respiratory syndrome (SARS)-coronavirus 2 (CoV-2) infection (“Coronavirus Disease 2019” [COVID-19]) within 7 days of planned start of study therapy. The use of all COVID-19 vaccines was allowed, with the exception of COVID-19 vaccines using the recombinant adenoviral vector platform within 30 days of planned start of study therapy. If a COVID-19 vaccine using this platform was to be administered within 30 days of planned start of study therapy, this must have been first discussed with and approved by the Sponsor’s Medical Monitor
- Patient had a known hypersensitivity to cobolimab components or excipients, or, if applicable, nivolumab, dostarlimab, or encelimab components or excipients
