# Supplementary material for: Combined Targeting of PD-1 and TIM-3 in Patients with Locally Advanced or Metastatic Melanoma: AMBER Cohorts 1c, 1e, and 2A
Source: Clin Cancer Res. 2025 Jun 24;31(16):3433–42. doi: 10.1158/1078-0432.CCR-25-0884 (PMC12351273; doi:10.1158/1078-0432.CCR-25-0884)
Supplement: Supplementary Figure S1 — AMBER Part 1c/1e and Part 2A design [file ccr-25-0884_supplementary_figure_s1_suppfs1.docx]

#### Figure S1. AMBER Part 1c/1e and Part 2A design


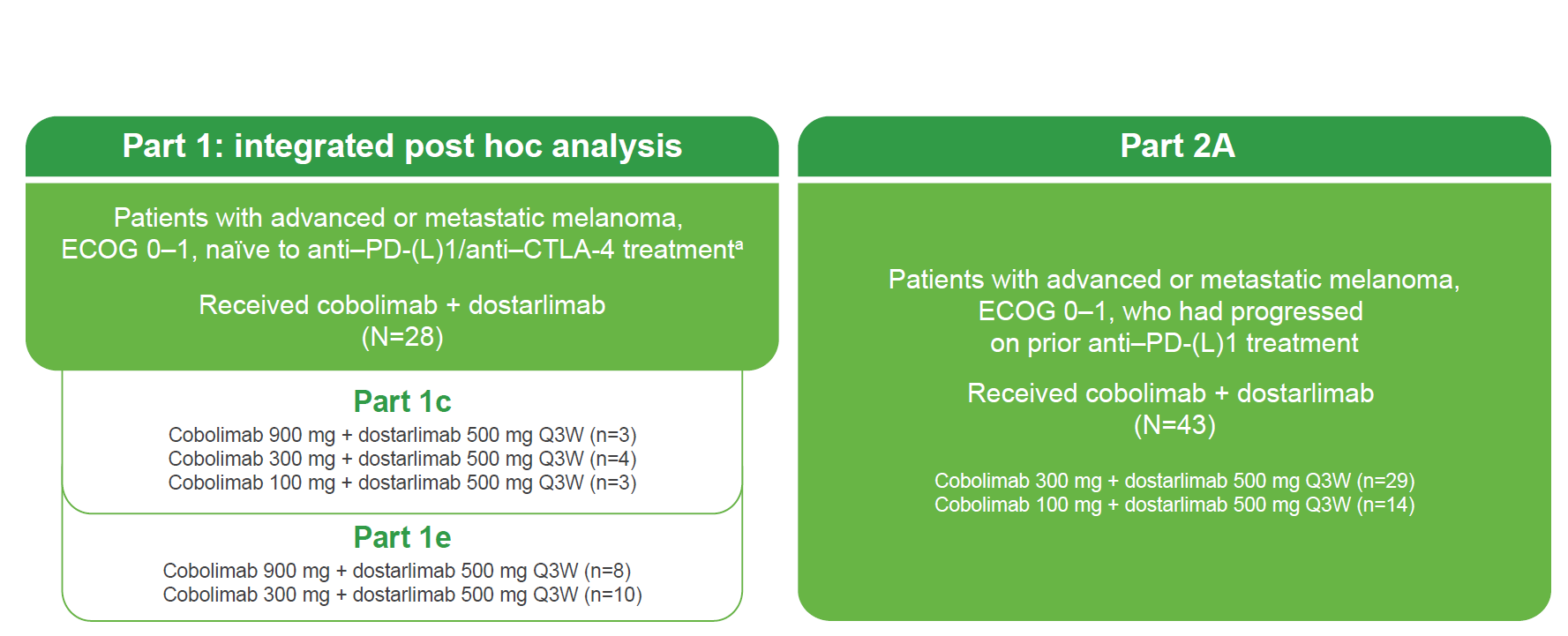


^a^BRAF-targeting therapies were permitted.

BRAF, v-raf murine sarcoma viral oncogene homolog B1; ECOG, Eastern Cooperative Oncology Group; CTLA-4, cytotoxic T-lymphocyte-associated protein-4; PD-(L)1, programmed cell death (ligand) protein-1; Q3W, every 3 weeks.
