# Supplementary material for: Combined Targeting of PD-1 and TIM-3 in Patients with Locally Advanced or Metastatic Melanoma: AMBER Cohorts 1c, 1e, and 2A
Source: Clin Cancer Res. 2025 Jun 24;31(16):3433–42. doi: 10.1158/1078-0432.CCR-25-0884 (PMC12351273; doi:10.1158/1078-0432.CCR-25-0884)
Supplement: Supplementary Figure S2 — Cobolimab binding to native TIM-3 in T cells and monocytes [file ccr-25-0884_supplementary_figure_s2_suppfs2.docx]

#### Figure S2. Cobolimab binding to native TIM-3 in T cells and monocytes


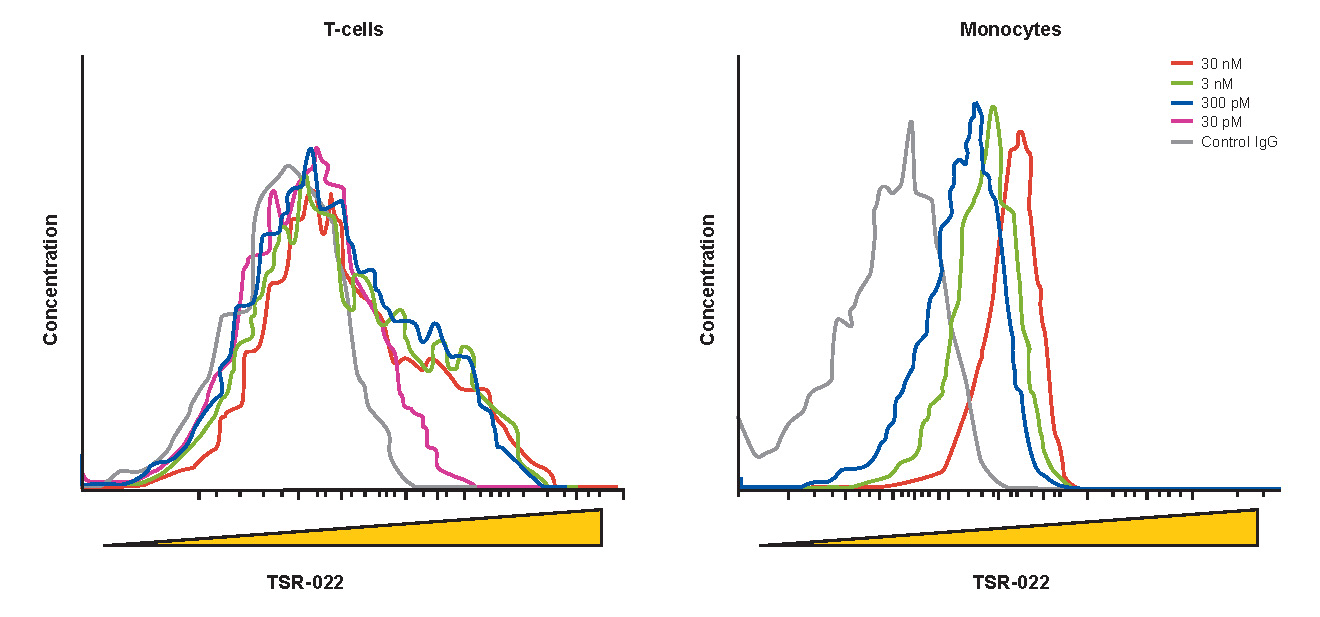

Ig, immunoglobulin; TIM-3, T-cell immunoglobulin and mucin-domain containing-3.
