# Supplementary material for: Combined Targeting of PD-1 and TIM-3 in Patients with Locally Advanced or Metastatic Melanoma: AMBER Cohorts 1c, 1e, and 2A
Source: Clin Cancer Res. 2025 Jun 24;31(16):3433–42. doi: 10.1158/1078-0432.CCR-25-0884 (PMC12351273; doi:10.1158/1078-0432.CCR-25-0884)
Supplement: Supplementary Table S1 — Cobolimab binding affinity [file ccr-25-0884_supplementary_table_s1_suppts1.docx]

#### Supplementary Table S1. Cobolimab binding affinity

|  | Kinetic parameters | | | TIM-3 expressing CHO cells | |
| --- | --- | --- | --- | --- | --- |
|  | K_assoc_ (Ms)^-1^ | K_dissoc_ (s^-1^) | K_D_ (pM) | EC_50_ | EC_90_ |
| Human | 1.5x10^7^ | 1.1x10^-4^ | 7 | 0.17 | 1.67 |
| Cynomolgus monkey | 1.1x10^7^ | 1.9x10^-4^ | 17 | 0.27 | 1.47 |

CHO, Chinese Hamster Ovary; EC_50/90_, concentration effective in producing 50/90% of the maximal response; K_assoc_, association rate constant; K_D_, equilibrium dissociation constant between the antibody and its antigen; K_dissoc_, dissociation rate constant; TIM-3, T-cell immunoglobulin and mucin-domain containing-3.
