# Supplementary material for: Combined Targeting of PD-1 and TIM-3 in Patients with Locally Advanced or Metastatic Melanoma: AMBER Cohorts 1c, 1e, and 2A
Source: Clin Cancer Res. 2025 Jun 24;31(16):3433–42. doi: 10.1158/1078-0432.CCR-25-0884 (PMC12351273; doi:10.1158/1078-0432.CCR-25-0884)
Supplement: Supplementary Table S2 — Representativeness of study patients [file ccr-25-0884_supplementary_table_s2_suppts2.docx]

#### Supplementary Table S2. Representativeness of study patients

| **Cancer type(s)/ subtype(s)/ stage(s)/ condition** | Melanoma |
| --- | --- |
| **Conditions related to:** | |
| **Sex** | Globally, metastatic melanoma is more common in men than women (1). SEER data indicates the age-adjusted incident rate for melanoma is higher in men than in women for all age groups; however, incidence for women under 20–49 is increasing (2). |
| **Age** | Melanoma risk generally increases with age (3), with a mean age of diagnosis of 65 years (4); however, *BRAF*-mutant melanoma is associated with a younger age at diagnosis (54 years) compared with wild-type melanoma (5). |
| **Race/Ethnicity** | The incidence of melanoma is higher in White patients versus patients of other races (4). In the United States, Hispanic, American Indian, and Asian patients are typically diagnosed at a later stage than White patients, and Black patients have a higher risk of mortality than White patients (6). |
| **Geography** | In 2020, the highest incidence rates were reported in Australia and New Zealand, followed by Western Europe and North America (7). |
| **Other considerations** | An estimated 75% of melanoma cases can be attributed to UV radiation (7), and the molecular profile of these mutations contributes to prognosis and response to treatment (4). |
| **Overall representativeness in this study** | The median age (65.0 years) and sex distribution of patients (58% male) in this study are in line with that reported in the literature.  This study recruited patients from the United States of America, Spain, Korea, and Taiwan. The majority of patients (89%) were White.  Genetic information was collected and this included genetic information for oncogenic drivers. |
| SEER, Surveillance, Epidemiology, and End Results.  1. Di Carlo V, Eberle A, Stiller C, Bennett D, Katalinic A, Marcos-Gragera R*, et al.* Sex differences in survival from melanoma of the skin: The role of age, anatomic location and stage at diagnosis: A CONCORD-3 study in 59 countries. *Eur J Cancer* 2025;**217**:115213 doi 10.1016/j.ejca.2024.115213.  2. Liu F, Bessonova L, Taylor TH, Ziogas A, Meyskens Jr FL, Anton‐Culver H. A unique gender difference in early onset melanoma implies that in addition to ultraviolet light exposure other causative factors are important. *Pigment Cell Melanoma Res* 2013;**26**(1):128-35 doi 10.1111/pcmr.12035.  3. Velazquez AI, Brewer JD. The epidemiology of melanoma in young adults. *Expert Rev Dermatol* 2013;**8**(6):707-16 doi 10.1586/17469872.2013.844465.  4. Saginala K, Barsouk A, Aluru JS, Rawla P, Barsouk A. Epidemiology of melanoma. *Med Sci (Basel)* 2021;**9**(4):63 doi 10.3390/medsci9040063.  5. Ribero S, Stucci L, Marra E, Marconcini R, Spagnolo F, Orgiano L*, et al.* Effect of age on melanoma risk, prognosis and treatment response. *Acta Derm Venereol* 2018;**98**(7):624-9 doi 10.2340/00015555-2944.  6. Cormier JN, Xing Y, Ding M, Lee JE, Mansfield PF, Gershenwald JE*, et al.* Ethnic differences among patients with cutaneous melanoma. *Arch Intern Med* 2006;**166**(17):1907-14 doi 10.1001/archinte.166.17.1907.  7. Arnold M, Singh D, Laversanne M, Vignat J, Vaccarella S, Meheus F*, et al.* Global burden of cutaneous melanoma in 2020 and projections to 2040. *JAMA Dermatol* 2022;**158**(5):495-503 doi 10.1001/jamadermatol.2022.0160. | |
